# Network Based Statistical Analysis Detects Changes Induced by Continuous Theta-Burst Stimulation on Brain Activity at Rest

**DOI:** 10.3389/fpsyt.2014.00097

**Published:** 2014-08-05

**Authors:** Chiara Mastropasqua, Marco Bozzali, Viviana Ponzo, Giovanni Giulietti, Carlo Caltagirone, Mara Cercignani, Giacomo Koch

**Affiliations:** ^1^Neuroimaging Laboratory, IRCCS Santa Lucia, Rome, Italy; ^2^Department of Neuroscience, Trieste University, Trieste, Italy; ^3^Department of Clinical and Behavioural Neurology, IRCCS Santa Lucia, Rome, Italy; ^4^Department of Neuroscience, University of Rome Tor Vergata, Rome, Italy; ^5^Clinical Imaging Sciences Centre, Brighton and Sussex Medical School, University of Sussex, Falmer, UK

**Keywords:** functional connectivity, cTBS, resting state fMRI, dorsolateral prefrontal cortex, fronto-parietal network

## Abstract

We combined continuous theta-burst stimulation (cTBS) and resting state (RS)-fMRI approaches to investigate changes in functional connectivity (FC) induced by right dorsolateral prefrontal cortex (DLPFC)–cTBS at rest in a group of healthy subjects. Seed-based fMRI analysis revealed a specific pattern of correlation between the right prefrontal cortex and several brain regions: based on these results, we defined a 29-node network to assess changes in each network connection before and after, respectively, DLPFC–cTBS and sham sessions. A decrease of correlation between the right prefrontal cortex and right parietal cortex (Brodmann areas 46 and 40, respectively) was detected after cTBS, while no significant result was found when analyzing sham-session data. To our knowledge, this is the first study that demonstrates within-subject changes in FC induced by cTBS applied on prefrontal area. The possibility to induce selective changes in a specific region without interfering with functionally correlated area could have several implications for the study of functional properties of the brain, and for the emerging therapeutic strategies based on transcranial stimulation.

## Introduction

Brain connectivity has been non-invasively assessed in human subjects using techniques focused on three general network properties: anatomical connectivity, functional connectivity (FC), and response to perturbation/stimulation (1).

Resting state (RS) fMRI is becoming one of the most popular techniques for assessing FC at rest (2, 3), while non-invasive brain stimulation methods can be used to probe how brain connectivity varies in response to an external perturbation. The combination of these techniques holds great promise for addressing important clinical issues (4–6).

Different approaches have been used to investigate the effect of a perturbation on fMRI FC; some studies have been performed to assess the influence of tDCS on RS-fMRI data (7–10). Other work focused on the influence of TMS on task-based effective connectivity (11–14). Just few studies investigated so far the effects of repetitive TMS (rTMS) on RS-fMRI. In one study, rTMS was applied over the left dorsolateral prefrontal cortex (DLPFC), resulting in distal changes of neural activity within the default mode network (DMN) (15). Similarly, two different frequencies of rTMS applied over the left posterior inferior parietal lobule (IPL) were tested to evaluate the effect on the DMN: high-frequency rTMS decreased functional correlations between cortical DMN nodes, but not between these nodes and the hippocampal formation. In contrast, low frequency rTMS increased functional correlations between IPL and the hippocampal formation (16). Another study tested the effects of rTMS on prefrontal–hippocampal coupling during both a working memory task and at rest. Seeded FC analyses demonstrated significant effects of rTMS on the prefrontal network dynamics in the *n*-back task that were not evident during rest (17). All these studies were performed with a strong working hypothesis, either testing only one resting state network (RSN) (15, 16) or using coupling analyses within a specified connection (17). Moreover, all of them compared the effects of rTMS in two separate sessions performed on different days, which could have increased the intrinsic variability of the FC measured by fMRI.

Here, for the first time we compared RS-fMRI data recorded before and after real continuous theta-burst repetitive stimulation (cTBS), a powerful protocol, resulting in long-lasting decreases of cortical excitability (18). In contrast with previous studies, we conducted a novel network based statistics (NBSs) (19) approach to include all the most relevant nodes of the areas interconnected with the stimulated site (the right DLPFC). We chose to stimulate the right DLPFC, since TMS of this area is known to modulate several cognitive functions and has a potential role in treating various clinical conditions (20, 21).

## Materials and Methods

The study was approved by the ethics committee of Santa Lucia Foundation, and written informed consent was obtained from all subjects before study initiation.

**Figure 1:**
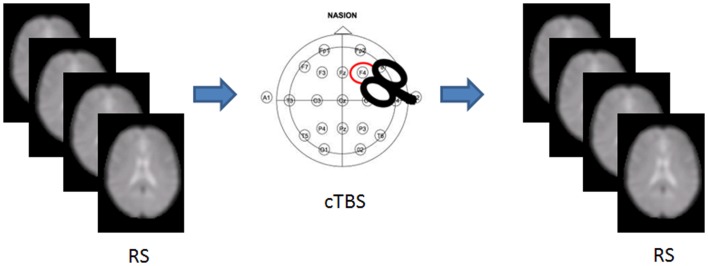
**Experimental set up**. RS-fMRI was acquired before and after cTBS stimulation in half of the participants.

We recruited 36 healthy volunteers [*m*/*f*  = 18/18; mean (SD) age = 26.88 (3.5) years] with no history of medical or psychiatric disorders, autonomic dysfunction, or other major clinical conditions. The experimental session included an MRI scan, followed by either cTBS or sham stimulation, and a post-intervention MRI scan (Figure 1). Each participant was randomly assigned to either group, resulting in 18 participants receiving cTBS, and 18 receiving the sham.

### MRI acquisition protocol

All imaging was obtained using a head-only 3.0 T MR scanner (Siemens Magnetom Allegra, Siemens Medical Solutions, Erlangen, Germany). The acquisition protocol included the following sequences: (1) a magnetization-prepared rapid gradient echo (MPRAGE) sequence (TR = 2500 ms; TE = 2.74 ms; TI = 900 ms; flip angle = 8°; matrix = 256 × 208 × 176; slab thickness = 1 mm; FOV = 256 mm × 208 mm × 176 mm). (2) A series of T2* weighted echo planar imaging (EPI) scans, sensitized to blood oxygenation level dependent contrast (BOLD) (TR = 2080 ms; TE = 30 ms; 32 axial slices parallel to AC–PC line; matrix = 64 × 64; pixel size = 3 mm × 3 mm; slice thickness = 2.5 mm; flip angle = 70°) for RS-fMRI. BOLD EPIs were collected during rest for a 7 min and 20 s period, resulting in a total of 220 volumes.

### cTBS protocol

A MagStim Super Rapid magnetic stimulator (MagStim Company, Whitland, Wales, UK), connected with a figure-of-eight coil with a diameter of 90 mm was used to deliver cTBS over the scalp site corresponding to the right prefrontal cortex (F4 electrode International 10–20 system). The magnetic stimulus had a biphasic waveform with a pulse width of about 300 μs. Three-pulse bursts at 50 Hz repeated every 200 ms for 40 s were delivered at 80% of the active motor threshold (AMT) over right DLPFC (600 pulses). AMT was tested over the motor cortex of the right hemisphere. AMT was defined as the lowest intensity that produced MEPs of >200 μV in at least 5 out of 10 trials when the subject made a 10% of maximum contraction using visual feedback (22).

Dorsolateral prefrontal cortex was targeted using a neuronavigation system (SofTaxic) to precisely position the coil over the cortical site, using individual T1-weighted magnetic resonance imaging volumes as anatomical reference; this technique has been previously described in detail (23, 24). The stimulation points were determined before the experiment and were marked on the adherent plastic cap worn by the subject. To target DLPFC, the coil was positioned over the middle of the line separating the anterior and middle thirds of this gyrus, following the algorithm proposed by Mylius and collaborators (25). According to the anatomical data reported by Rajkowska and Goldman-Rakic (26), this target is localized at the junction between BA9 and BA46. This location is in agreement with meta-analyses of neuroimaging studies on working memory (27, 28). The center of the coil was positioned tangentially to the skull with the handle pointing backward angled at 45° (Figure 1). For sham cTBS, the coil was positioned over the same scalp site, but angled away so that no current was induced in the brain.

### fMRI pre-processing

The RS-fMRI data were processed using MATLAB R2007B (Math-Work, Natick, MA, USA) and SPM8^1^. The first four volumes of the functional images were discarded for signal equilibrium and adaptation of participant to scanning noise. Next, slice timing and head motion correction were performed. Participants exhibiting head motion of >2 mm maximum translation of 2° rotation throughout the course of scan were excluded. The images were then normalized using the EPI template provided with SPM8.

In-house software was used to remove the global temporal drift using a third order polynomial fit, the realignment parameters, and the signal averaged over whole brain voxels. Data were band-pass filtered (between 0.01 and 0.08 Hz).

### Seed-based connectivity analysis

Seed-based connectivity analysis (SBA) was performed to identify the cortical areas functionally connected with the stimulated region. For each subject the mean time course (TC) of the right DLPFC was extracted for each subject using the prefrontal cortex region defined in Harvard Oxford atlas^2^, available with FSL.

Each participant’s TC was then used as regressor in a first-level analysis in SPM8, in order to identify the degree of correlation, for every voxel in the brain, with the prefrontal region, adjusting for the motion parameters. Contrast images for positive correlation were fed into a second level analysis using a one-sample *T*-Test. Results were considered significant for *p* < 0.05 FWE corrected at voxel level.

### Network based statistic

**Figure 2:**
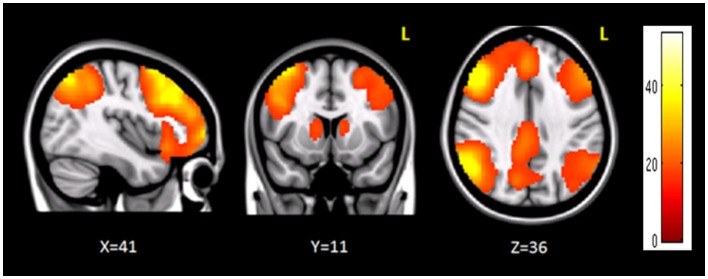
**Brain regions functionally correlated to the right prefrontal cortex used as seed in seed-based analysis**. The color bar represents the *t*-scores.

**Figure 3:**
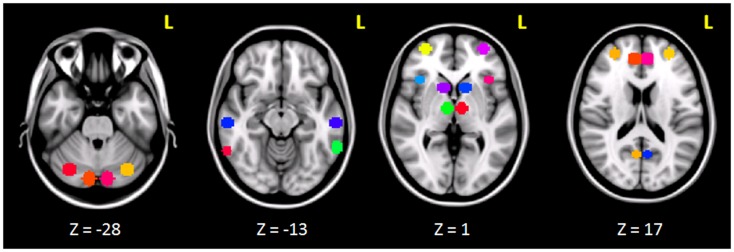
**Spherical ROIs (radius = 8mm) defining the nodes of the network investigated before and after cTBS**.

The clusters that resulted to be significantly connected to the right prefrontal cortex (Figure 2) were then defined as the nodes of the network of interest. Using MarsBaR^3^, we created 29 spheres, with a diameter of 8 mm each (see Table 1; Figure 3), centered at the center of gravity of each of the nodes, from which mean TCs were extracted to estimate a connectivity matrix for each subject. The number of rows and columns in this matrix is the total number of nodes in the network, and the elements are defined as the correlation coefficient between the TC of each pair of nodes.

**Table 1:** **The table shows the regions used to create the 29-sphere network, their corresponding Brodmann area and MNI coordinates of the center of each sphere**.

Region	Brodmann area	*X*	*Y*	*Z*
Cingulate gyrus (posterior division)		2	−34	40
**RIGHT**				
Frontal pole	BA10	32	56	6
Intracalcarine cortex	BA17	6	−62	12
Middle temporal gyrus (posterior division)	BA20	60	−22	18
Paracingulate gyrus (anterior division)	BA32	8	44	20
Middle temporal gyrus (temporoccipital part)	BA37	62	−50	−10
Supramarginal gyrus (posterior division)	BA40	48	−44	50
Frontal pole	BA46	30	50	24
Frontal orbital cortex	BA47	38	22	−4
Precuneous cortex	BA7	8	−66	46
Middle frontal gyrus	BA9	38	24	46
Caudate nucleus		12	14	6
Cruz I (medial cerebellum)		10	−82	−28
Cruz II (lateral cerebellum)		32	−72	30
Thalamus		8	−10	6
**LEFT**				
Frontal pole	BA10	−32	56	6
Intracalcarine cortex	BA17	−6	−62	12
Middle temporal gyrus (posterior division)	BA20	−60	−22	18
Paracingulate gyrus (anterior division)	BA32	−8	44	20
Middle temporal gyrus (temporoccipital part)	BA37	−62	−50	−10
Supramarginal gyrus (posterior division)	BA40	−48	−44	50
Frontal pole	BA46	−30	50	24
Frontal orbital cortex	BA47	−38	22	−4
Precuneous cortex	BA7	−8	−66	46
Middle frontal gyrus	BA9	−38	24	46
Caudate nucleus		−12	14	6
Cruz I (medial cerebellum)		−10	−82	−28
Cruz II (lateral cerebellum)		−32	−72	30
Thalamus		−8	−10	6

*Every sphere had an 8 mm radius*.

Once each participant connectivity matrix was obtained, we used the NBS toolbox (19)^4^ to compare the correlation between each node of the network before and after cTBS, using a paired *T*-test design. The false discovery rate (FDR) was used to adjust for multiple comparisons, with 25,000 permutations. Results were considered significant for *p* < 0.05. The same analysis was performed on the data acquired before and after sham stimulation.

## Results

Four participants who received sham stimulation were excluded due to excessive motion during fMRI, thus resulting in the following two groups: 18 subjects receiving cTBS [*m*/*f*  = 9/9; mean (SD) age = 26.72 (3.8) years] and 14 receiving sham stimulation [*m*/*f*  = 6/8; mean (SD) age = 27.07(3.6) years].

**Figure 4:**
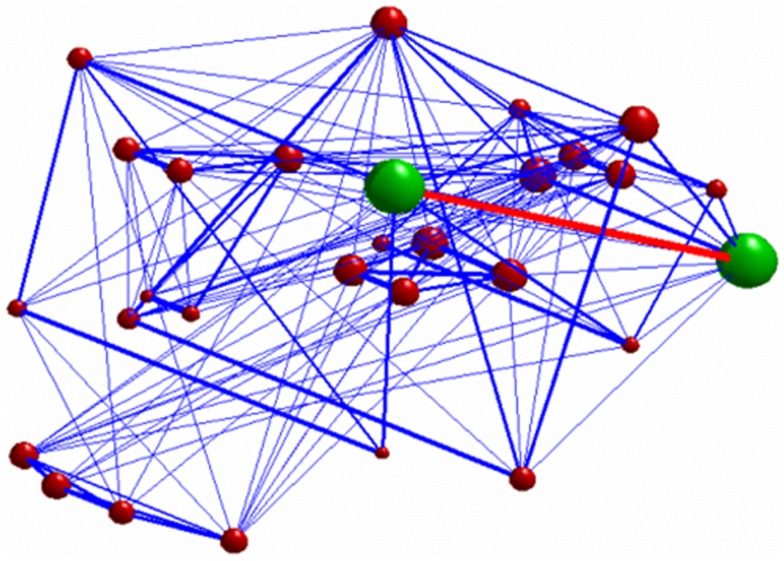
**3D graph representing the investigated network**. The green nodes indicate the ROIs whose connectivity (represented by the red edge) was decreased after stimulation. The radius of each of the red nodes reflects the node strength (i.e., the sum of the weights of each edge connected to the node). The thickness of the edges reflect the strength of correlation between each node. Only connections with correlation coefficient >0.3 are displayed.

**Figure 5:**
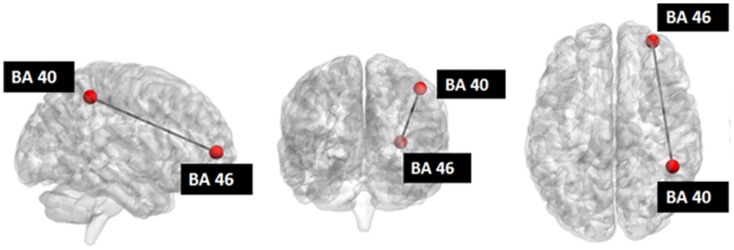
**Investigated network: the red nodes indicate the ROIs whose connectivity was decreased after stimulation**.

Seed-based connectivity analysis revealed a specific pattern of correlation between right DLPFC and several brain regions, including the right and left prefrontal, parietal, temporal cortex, precuneus, posterior cingulated cortex, thalamus, caudate nucleus, and cerebellum (Figure 2). The corresponding network nodes are shown in Table 1 and Figure 3. We detected a striking decreased correlation between the right DLPFC and the right posterior parietal cortex (Brodmann areas 46 and 40, respectively) after stimulation (*p* < 0.05) (Figures 4 and 5). The same analysis performed on the data acquired before and after sham stimulation did not show any difference among the tested connectivity matrices.

## Discussion

In this study, we provide new evidence for the role of RS-fMRI in detecting changes in brain activity associated with TMS. Through RS-fMRI it is possible to identify functional connections that reflect temporal coupling between distant regions. Thus, characterizing the covariance of the BOLD signal in anatomically distant areas of the brain can be useful to measure the degree to which the network properties are affected by TMS. Here, RS-fMRI was carried out before and immediately after TMS to provide direct measures of the functional organization of the DLPFC-correlated network and its plastic reorganization induced by stimulation.

To assess the influence of the perturbation induced by TMS on FC, we used NBS. It is a novel network based approach to identify functional correlations between different brain regions known to be part of a specific pattern of co-activation. This methodology is based on graph theory, which provides a theoretical framework to examine complex networks, thus revealing important information about their local and global organization (5, 16). NBS was used after the identification of a specific network of right DLPFC-correlated regions, in order to restrict the analysis to the nodes showing functional connections to the stimulation site. Such network was identified using SBA, and it strictly resembles a network previously described as the right fronto-parietal network (FPN) by several groups (5, 29–32). Accordingly, we found that DLPFC cTBS induced a selective modulation of the ipsilateral posterior parietal cortex. This finding could be interpreted on the basis of the well known functional interactions strongly linking the activity of the DLPFC with that of the PPC (33). These two areas are jointly implicated in a variety of cognitive functions and are thereby considered two main nodes of the FPN. Indeed, throughout the literature, two strongly lateralized RSNs have consistently been reported, one predominantly in the right hemisphere and the other in the left hemisphere usually with a specular pattern involving the middle frontal and orbital cortex (BA 6/9/10), the superior parietal cortex (BA 7/40), the middle temporal gyrus (BA 21), and the posterior cingulate cortex (BA 23/31) (30). These two networks are known to be closely coupled in a wide range of cognitive processes, such as working memory, both in adults (30, 34–36) and in children and adolescents (37–39), language (40), attention (41–44), and visual processes (45).

Consistently, recent tDCS literature suggests that low-intensity electrical stimulation over the DLPFC can result in transient improvements in a variety of cognitive functions including declarative (46) and working memory (47, 48), planning (49), language learning (46), attention (50), and decision making (51).

To better understand the substrate of these changes, the interaction between the nodes of the FPNs has been investigated using tDCS. A previous study (8) examined how active tDCS over the left or right DLPFC in comparison with sham tDCS modulates TC fluctuations within and across the DMN and the anti-correlated network (AN) on RS-fMRI. One of the main results emerging from this work is that active anodal tDCS over the DLPFC results in a stronger temporal FC between prefrontal and parietal regions, supporting our current findings. Similar results were obtained by Keeser et al. (7), who measured significant changes in regional brain connectivity for nodes of the DMN and the right and left FPNs. Such changes were detected after DLPFC–tDCS both, close to the primary stimulation site, and in connected brain regions.

On the other hand, the effects of TMS (as opposed to tDCS) have been mainly evaluated in combination with task-active fMRI, instead of RS-fMRI. Only recently, a number of studies attempted to assess the effect of TMS on FC at rest (11, 13–16, 52, 53). Most of these works were performed with a strong working hypothesis, either testing only one RSN (15, 16) or using coupling analyses within a specified connection (17). Crucially, all of them evaluated the effects of rTMS by comparing post-stimulation vs. post-sham data recorded on separate days, introducing a bias due to the intrinsic variability of RS-fMRI. In contrast, in order to reduce the effect of intrinsic individual variability, we compared for the first time RS-fMRI data recorded within the same session before and after TMS, with a short interval between MRI sessions.

Our data indicate a selective influence of right DLPFC–cTBS on the ipsilateral posterior parietal cortex, while no connectivity change was detected after sham stimulation. As it is known that cTBS is able to induce prolonged cortical inhibition (18), the decreased correlation between BA46 and BA40 we observed after stimulation could be explained by two alternative hypotheses: (i) cTBS is able to induce cortical inhibition just in the stimulated site with a consequent disruption of the co-activation of the two areas; (ii) the inhibition of cortical activity occurs immediately in the stimulation site, subsequently spreading to distant connected area. The propagation of inhibitory signal at microscopic level induces a de-synchronization of normal coupling activity of the areas involved. So the de-coupling of neuronal activity we observed through the BOLD signal could reflect an undergoing mechanism of signal propagation. Thus, we hypothesize that cTBS does not solely produce focal effects by selectively affecting an isolated patch of cortex. Rather, target sites should be considered as nodes within a widespread network of interacting brain regions, where perturbing or boosting processing of one element can also influence several others. We can only speculate on why we found a selective modulation of the DLPFC–PPC connection. The DLPFC and PPC neuronal assemblies have a strong functional coupling that could be more sensible to an external perturbation such as that induced by the low-intensity cTBS protocol applied in the current study (33). However, it is likely that by simply increasing the intensity of the magnetic field or changing the frequency of stimulation it could be possible to affect the coupling among other interconnected nodes. Notably, recent evidence suggests that an individual approach based on FC MRI could provide the most reliable approach to detect the effects of DLPFC TMS (1, 54).

Our results could also have several implications for clinical applications, as it has been demonstrated the role of rTMS of the DLPFC in the treatment of major depressive disorder (MDD). This therapeutic effect can be achieved by either excitatory stimulation of the left (52, 55–57) or inhibitory stimulation of the right DLPFC (58–60). A recent meta-analysis study conducted by Chen and collaborators (20), demonstrated that, despite the comparable efficacy of both methodology, the latter (inhibitory TMS) may be a more acceptable treatment for MDD than the former (excitatory TMS), based on patients reporting less headaches, and on the decrease risk of inducing adverse events such as seizures (61). The present results could also be important for other conditions in which the non-invasive modulation of the FPN can provide notable clinical improvements, such as the case of post-stroke hemispatial neglect (62).

In conclusion, our findings provide new insights into the mechanisms of stimulation-induced brain plasticity by demonstrating that the network communication at rest shapes the brain reorganization induced by cTBS. The use of TMS and RS-fMRI allows to characterize both local (i.e., in the cortical tissue directly under the TMS coil) and remote (i.e., distant from the original cortical target site) effects of TMS in more detail, leading to a better understanding of TMS-induced modulations in neural processing.

## Conflict of Interest Statement

The authors declare that the research was conducted in the absence of any commercial or financial relationships that could be construed as a potential conflict of interest.

## Footnotes

^1^http://www.fil.ion.ucl.ac.uk/spm/software/spm8/

^2^http://www.cma.mgh.harvard.edu/

^3^http://marsbar.sourceforge.net/

^4^http://www.cmtk.org/viewer/documentation/users/tutorials/tut_nbs.html
